# Evaluation of the efficacy of hydroxyl radical (OH˙) release for disinfection of the air and surfaces in the dental clinic: an *in vitro* study

**DOI:** 10.4317/medoral.26157

**Published:** 2023-07-10

**Authors:** Anais Paños-Crespo, Jorge Toledano-Serrabona, María Ángeles Sánchez-Garcés, Cosme Gay-Escoda

**Affiliations:** 1 DDS, MS. School of Medicine and Health Sciences, Campus de Bellvitge, University of Barcelona. Barcelona, Spain; 2 DDS, MS, PhD. Professor of Oral Surgery. School of Medicine and Health Sciences, Campus de Bellvitge, University of Barcelona. Researcher of the IDIBELL Institute, Barcelona, Spain; 3 MD, DDS, MS, PhD, EBOS. Professor of Oral Surgery. School of Medicine and Health Sciences, Campus de Bellvitge, University of Barcelona. Researcher of the IDIBELL Institute, Barcelona, Spain; 4 MD, DDS, MS, PhD, EBOS, OMFS. Chairman of Oral and Maxillofacial Surgery. School of Medicine and Health Sciences, Campus de Bellvitge, University of Barcelona. Director of the Master in Oral Surgery and Buccofacial Implantology (EFHRE International University / FUCSO). Coordinator / Researcher of the IDIBELL Institute. Head of the Department of Oral Surgery, Buccofacial Implantology and Maxillofacial Surgery. Teknon Medical Center, Barcelona, Spain

## Abstract

**Background:**

Concerning about the quality of room air has increased exponentially. Specially in dental clinics where diary practice is characterized by the important generation of aerosols.

**Material and Methods:**

An *in vitro* model was used in which samples were collected from the surfaces and room air of a dental clinic before and after the use of an OH˙ radical generator.

**Results:**

A total of 1260 samples were collected for bacteriological analysis and 14 samples for the detection of SARS-CoV-2. Following OH˙ treatment, the tested surface samples showed a decrease in the number of colony forming units (CFUs) of 76.9% in TSA culture medium. The circulating room air samples in turn showed a decrease in CFUs of 66.7% in Sabouraud medium and 71.4% in Mannitol agar medium. No presence of SARS-CoV-2 was observed on the surface of the face shield.

**Conclusions:**

The disinfectant technology based on the use of hydroxyl radicals (OH˙) is effective in reducing the presence of moulds and yeasts and *Staphylococcus* in the air, and in reducing total aerobic bacteria on the tested surfaces.

** Key words:**Disinfection methods, hydroxyl radical, environment, surfaces, dentistry.

## Introduction

The study of pathogenic microorganisms and of methods to avoid their spread has become the focus of many research projects, particularly since the start of the COVID-19 pandemic (1). Efforts to guarantee correct disinfection and sterilization of the dental clinic should consider all types of organisms, since the oral cavity is characterized by the presence of biofilm containing over 700 microbiological species from the mucous membranes and saliva (2). In relation to bacteria, mention must be made of grampositive organisms such as the genus *Streptococcus* and *Staphylococcus aureus*, while most nosocomial fungal infections are attribuTable to the genus *Candida*. In turn, the most frequent cross-infections due to viruses include human immunodeficiency virus (HIV)(Retroviridae), hepatitis B and C viruses (Hepadnaviridae and *Flaviviridae*, respectively), herpes simplex virus (HSV) 1 and 2 (Herpesviridae), Varicella-Zoster virus (VZV)(Herpesviridae) and Epstein-Barr virus (EBV)(Herpesviridae), among others (2).

Traditionally, the main surface disinfectants used in the dental clinic have been chemical agents such as quaternary ammonia, alcohols, formaldehyde, hypochlorites or iodinated solutions (1,3). However, in the dental clinic, where abundant aerosols are formed, some pathogens may remain suspended in the air for a variable period of time, particularly in closed areas with poor ventilation (4-6). In this regard, the guidelines referred to air conditioning and purification in the dental clinic recommend the use of an air ventilation and/or purification system capable of guaranteeing a renewal of 6 volumes/hour (7). The most widely used methods are based on chlorine dioxide, ozone (O3), ultraviolet radiation (UV) and HEPA (High Efficiency Particulate Air) filters (8,9). However, despite the range of options in this field, no studies have compared their efficacy and advantages or side effects and risks.

On the other hand, techniques have been developed based on the release of hydroxyl radicals (OH˙), which constitute the most important natural oxidant agent as an Open Air Factor effect. Their bactericidal action is mediated by an advanced oxidation processes (AOP) that takes place in the membranes (lipids), proteins (amino acids) and genetic material (RNA and DNA nucleotides) of the pathogens. These techniques are able to destroy the great majority of pathogens at a concentration of 0.8 mg/l, i.e., using the equivalent of one ten thousandth of the required dose of conventional chemical disinfectants, with a processing time of four seconds and without producing cytotoxic (harmful) residual compounds. The hydroxyl radical emissions and dispersion capacity through natural chain reaction at RH 50% and 23ºC are from 14,21*106 molecule cm3 per second and 43,56* 106 molecule cm3 per second, very far from the natural emissions (10) that we breath abroad and equivalent to one thousandth of that of other disinfectants (11-12). This implies two major advantages: the dental professionals are able to continue working while the agent is being used, and it can be employed in large spaces. In other words, OH˙ generators, along with high pressure or negative pressure systems combined with HEPA filters, appear to offer good results and have been postulated as a primary disinfection strategy (2,7,9).

The present study constitutes the first experimental *in vitro* investigation of the efficacy of OH˙ in disinfecting the surfaces and room air of dental clinics.

## Material and Methods

The present *in vitro* study was divided into two phases (Ia and Ib) and involved the use of a hydroxyl radical (OH˙) releasing system based on hydrogen peroxide (WellisAir Disinfection Wadu02®- Airtècnics, Castellar del Vallès, Barcelona, Spain). The device measures 220 mm in width, 150 mm in depth and 370 mm in height, with a weight of 1.9 kg and a power rating of 3.6 W/hour. It has no filters but contains a cartridge that generates hydrogen peroxide (H2O2) and must be replaced every three months. It is also equipped with a real-time sensor that reports air quality based on a range of colors, and a night mode in which the noise generated by the device is less than 30 dB.

**Figure 1:**
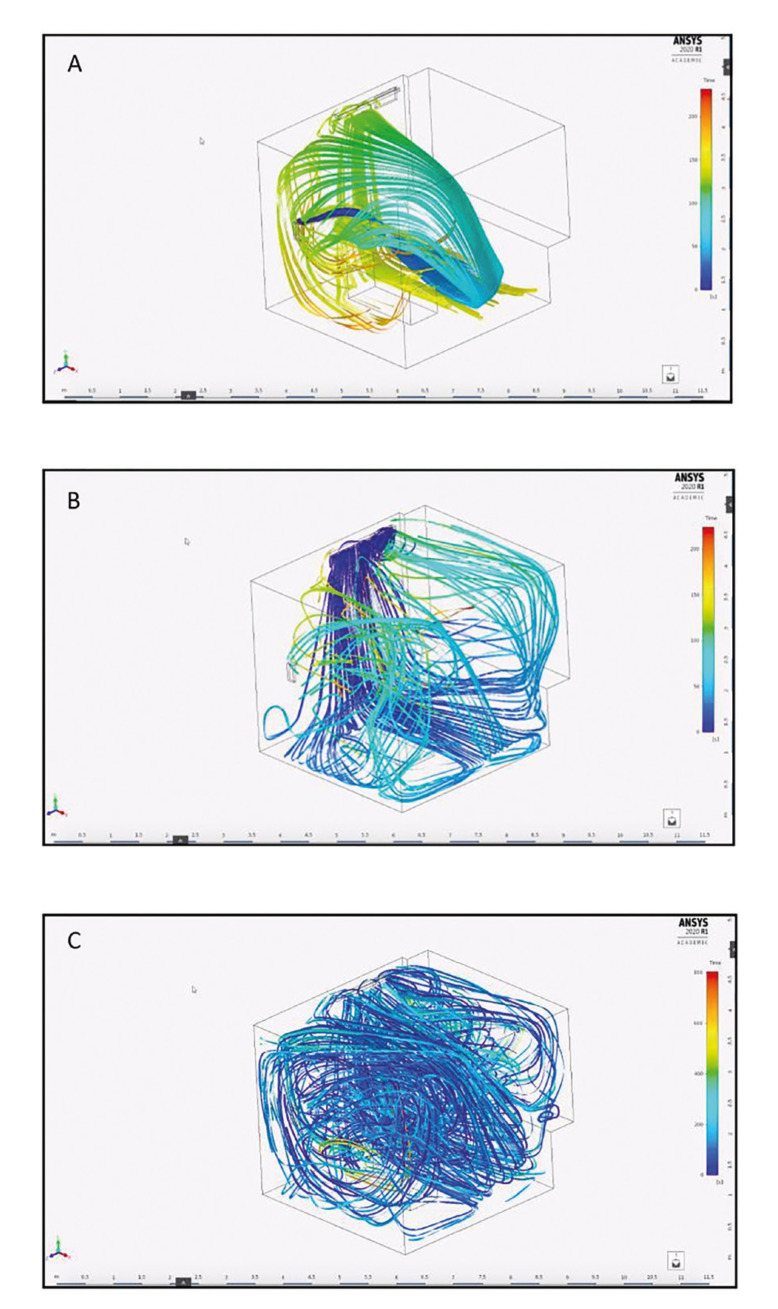
A) Flow lines of the hydroxyl radicals (OH˙) generated by the WellisAir Disinfection Wadu02® device. B) Flow lines of the vertical air conditioning impulsion grids. This profile covers the entire room and generates the trajectory of the hydroxyl radicals (OH˙). C) Mixing of the air conditioning with the hydroxyl radicals (OH˙) under stabilized conditions.

Study phase Ia was carried out in a dental office of Centro Medico Teknon (Barcelona, Spain), with a useful surface of 10.92 m2. Prior to the *in vitro* study, an analysis was made of the ventilation system, based on the geometric dimensions of the office, the different air conditioning flows, and air outlet and return. The circulating volumetric flow in the dental office was estimated from the total flow (20 m3/min) generated by the main air conditioning machine, taking into account the distribution of the flow lines (Fig. 1).

Phase Ia was carried out over 5 non-consecutive days (13, 15, 22 and 29 September, and 4 October, 2021), evaluating the disinfection capacity of OH˙ against bacteria on the surfaces and in the air of the dental office, and against SARS-CoV-2 on the face shield of the operator.

All the studied samples were obtained from the walls of the dental office, the protective face shield of the operator, the surface of the dental chair, and the room air. Samples that were contaminated or could not be analyzed were excluded. We collected control samples during the first two days prior to utilization of the OH˙ generating device. Then, the device was installed and the same sampling procedure was carried out for another three days, always maintaining the routine disinfection measures based on the use of a spray with 550 mg/g of ethanol, N,N-didecyl-N-methyl-polyoxyethyl ammonium propionate and 1.1 mg/g of perfume (Instrunet Inibsa® Spray, Laboratorios Inibsa S.A., Lliçà of Vall, Barcelona, Spain).

Sampling of bacteria from the surfaces of two walls of the dental office, the back and arm rest and fixed arm of the dental chair, and the protective face shield of the operator, was performed using RODAC plates in triplicate, with 7 different culture media (6 for bacteria and one for the presence of moulds and yeasts) (Table 1). The samples were sent to the Health and Environmental Microbiology Laboratory (MSMLab) of Barcelona Polytechnic University (UPC) for incubation and the posterior count of colonies.

For the detection of SARS-CoV-2, sampling was made of the protective face shield of the operator with an area of 20 x 100 cm2, following the recommended protocol for detecting the virus on industrial or hospital surfaces (Laboratorios Echevarne. División Industrial. Barcelona, Spain). The samples were kept refrigerated until processing with the reverse transcription polymerase chain reaction (RT-PCR) viral RNA test.

In the case of the air samples, three capturing devices were used in order to obtain samples from the entire office in triplicate at the same time. The devices containing the Petri plates were placed at a distance of at least one meter from the dental chair and at a height of 1.5 meters.

The samples were collected at three timepoints distributed over the start of the work day, at the end of a treatment, and after a dental procedure in which maximum aerosol production was expected to occur. All the treatment procedures performed on different days were equivalent in terms of the type of intervention and its duration. Specifically, 9 periodontal procedures with ultrasound and 6 dental treatments involving the use of a turbine were carried out.

Surface sampling for the detection of SARS-CoV-2 was carried out on the protective face shield of the operator. For this purpose we used a kit composed of two tubes: one containing a swab and the other containing saline solution.

**Table 1:**
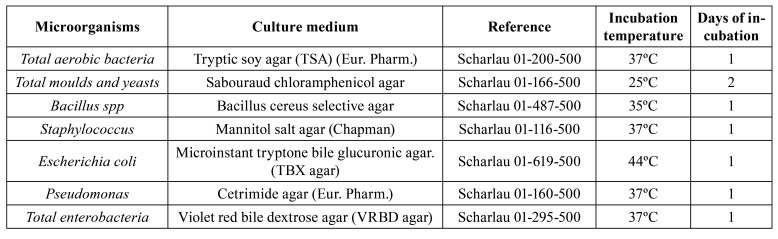
Culture media used for each of the microbial indicators

The tip of the swab was fully immersed in the saline solution tube, and the excess liquid was then drained by pressing the swab against the inner walls of the tube. The swab was subsequently used to sample the face shield, rotating and rubbing it in zigzag from one side to the other. This procedure was done twice: from left to right and then from top to bottom of the face shield. Lastly, the swab was returned to its tube, without liquid.

The primary study variable was the colony forming unit (CFU) count and the secondary study variable was the presence or absence of SARS-CoV-2 on the protective face shield of the operator. All data was recorded in a Microsoft Excel® (Microsoft Corp., Redmond, Washington, USA) and subsequently imported into STATA 14.2 software (StataCorp®, College Station, USA). For categorical variables, a descriptive analysis was carried out based on Tables of absolute and relative and bivariate frequency measurements with a Chi-square test or Fisher's exact test. Odds ratios (OR) with a 95% confidence interval (95% CI) were used as a measure of association. Normality of scale variables (CFU) were explored through Shapiro-Wilk’s test and visual analysis of the P-P and box plots. To analyze the effect of the device on the reduction of CFU an ANOVA of one factor was used for each of the culture media. In all cases, the significance level was set at *P*<0.05.

## Results

During phase Ia of the study we collected a total of 1260 samples for microbiological analysis and 14 samples for the detection of SARS-CoV-2 of the surface of the face shield of the operator. Likewise, a total of 200 liters of room air were sampled per Petri plate. Table 2 shows the total plates used for each of the culture media and sampled environments.

**Figure 2:**
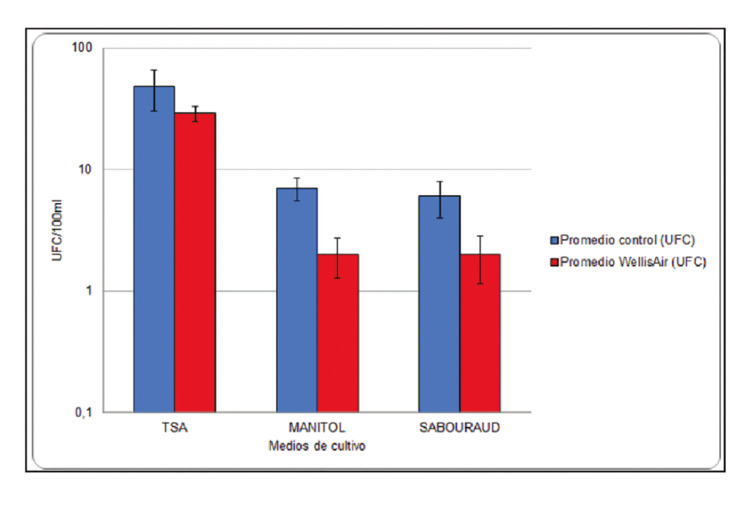
Effect of the hydroxyl radical (OH˙) releasing device in reducing total aerobic bacteria, <italic>Staphylococcus spp</italic>. (grampositive bacteria), moulds and yeasts.

The results referred to the air samples evidenced growth in Sabouraud agar for moulds and yeasts, tryptic soy agar (TSA) for total aerobic bacteria, and Mannitol for grampositive bacteria of the genus *Staphylococcus*, in the control and test samples (Fig. 2).

**Table 2:**
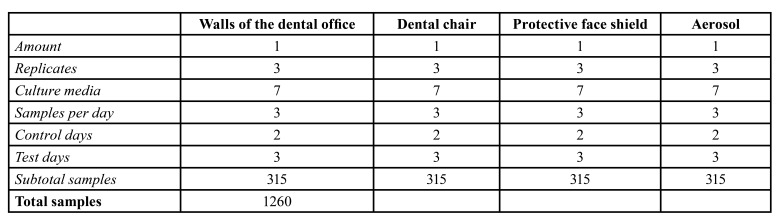
Number of plates used in sampling each environment. Total number of samples obtained after sampling three types of surface and the room air.

However, growth was absent in the control and test samples in 99% of the plates with tryptone bile X-glucuronide (TBX) medium for the gramnegative bacterium *Escherichia coli*, in cetrimide medium for *Pseudomonas*, in violet red bile dextrose (VRBG) agar for total enterobacteria, and in Mannitol egg yolk polymyxin (MYP) agar for* Bacillus spp*.

In TSA medium for total aerobic bacteria, the observed decrease in microbial growth was not statistically significant (*p* =0.65) in the test samples. In contrast, a significant decrease (66.7%) (*p*=0.03) was recorded in Sabouraud medium for moulds and yeasts in the test samples, and also in Mannitol agar for *Staphylococcus* (71.4%) (*p*=0.001).

**Figure 3:**
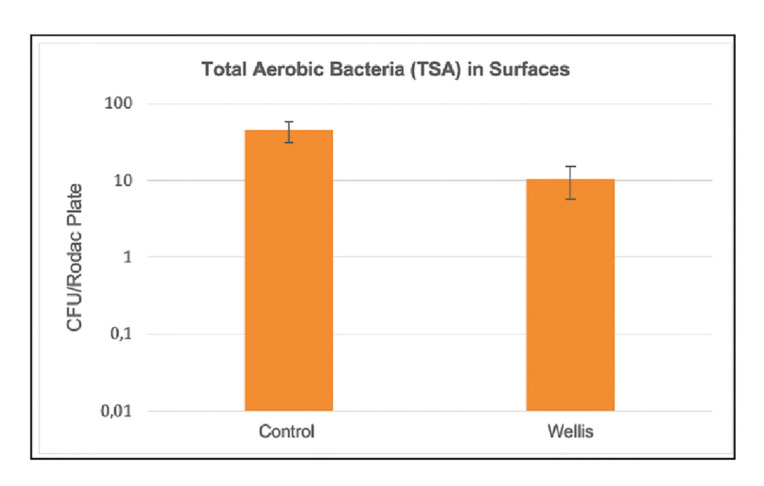
Effect of the hydroxyl radical (OH˙) releasing device in reducing total aerobic bacteria (TSA medium).

**Figure 4:**
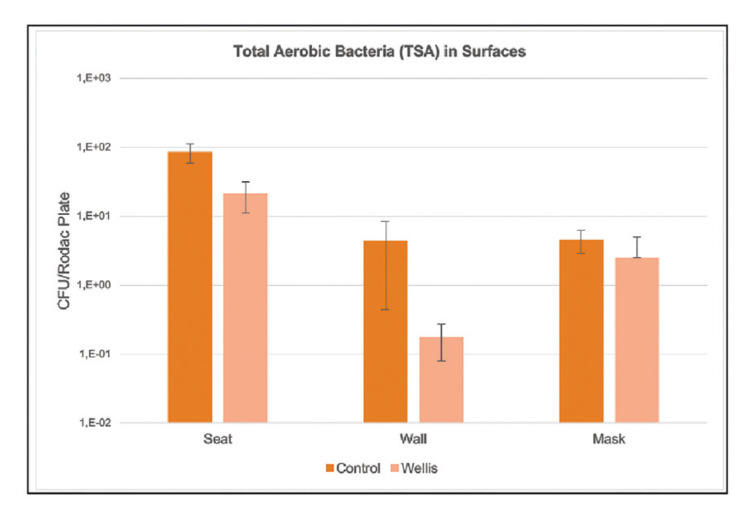
Effect of the hydroxyl radical (OH˙) releasing device in reducing total aerobic bacteria (TSA medium) on surfaces.

Regarding the results corresponding to the surfaces, no growth was observed (0 CFUs per RODAC plate) with the culture media for *Staphylococcus*, moulds and yeasts, *Escherichia coli*, total enterobacteria,* Bacillus spp*. and *Pseudomonas*. Growth was only observed with TSA medium for total aerobic bacteria (Fig. 3), with a decrease of 76.9% in the test samples (*p*=0.01). Of the different sampled surfaces, significant results were only recorded for the dental chair (*p*=0.02) (Fig. 4). The results corresponding to the walls of the dental office and the face shield of the operator failed to reach statistical significance (*p*=0.15 and *p*=0.27, respectively).

Lastly, no presence of SARS-CoV-2 was observed in phase Ia of the study in the 14 samples obtained from the surface of the face shield.

## Discussion

The choice of the 7 culture medias was made to assess the growth of the most common nosocomial pathogens in dental practice. However, CFUs were only quantified in the Sabouraud, Mannitol and TSA media.

With regard to moulds and yeasts, disinfection based on OH˙ release yielded a significant decrease exclusively in room air of about 67%. In comparison, in the study published by Moccia *et al*. (13), which evaluated the effect of an ozone (O3) releasing device using plate count agar (PCA) for the culture of mesophilic aerobic microorganisms and Sabouraud dextrose agar for moulds and yeasts, the observed reduction was over 90% for both surfaces and air. However, it is important to note that the use of ozone is not compatible with routine clinical practice, since its inhalation can result in serious lung problems and the appearance of skin irritation (13).

In relation to the genus *Staphylococcus*, we recorded a decrease of 71%, though only in the air samples. This percentage is greater than in the study by Wong *et al*. (14), where the Inov8® OH˙ releasing device produced a decrease in *Staphylococcus epidermidis* of 50-60%.

When compared with ultraviolet A (UV-A) radiation, OH˙ based disinfection offers a number of advantages, as demonstrated by Yamaguchi *et al*. (15), since UV-A exposure only resulted in a 40% decrease in the presence of *Staphylococcus aureus* on surfaces, and moreover the disinfection process had to carried out in the absence of both the healthcare professionals and the patients, due to the risk of adverse effects such as skin cancer or retinal photokeratitis. This does not happen with OH˙, which can be used concomitant to care activity.

In the case of total aerobic bacteria, the decrease in CFUs was only seen to be significant in the samples obtained from the dental chair. This can be explained by the direct contact with different patients in the course of the work day, despite the use of conventional antiseptics to disinfect the surfaces between patients.

In the present *in vitro* study, the WellisAir Disinfection Wadu02® OH˙ generating device produced reductions over 70% in the counts of *Staphylococcus*. However, grow was absent in the agar TBX for *Escherichia coli*. These are probably attribuTable to a number of factors. On one hand, the cell wall composition of the bacteria. In effect, grampositive pathogens (such as the genus *Staphylococcus*) have a thick cell wall composed mainly of peptidoglycans, while gramnegative bacteria (such as *Escherichia coli*) are characterized by a much thinner wall composed of phospholipids, lipopolysaccharides and lipoproteins. These structural differences make gramnegative bacteria more vulnerable to attack by reactive oxygen species (ROS) such as OH˙. On the other hand, our study was the first to perform sampling under non-controlled environmental conditions with the real presence and movement of people, reflecting daily practice in the dental clinic - though this also limited sampling homogeneity. Lastly, the microbiota count in the dental clinic was 1 Log10 (log units), which is lower than that found in the laboratory experiments. This can probably be attributed to the hygienization and chemical disinfection protocols commonly used in dental practice, and which explain why the observed count reductions were not more significant.

The fact that SARS-CoV-2 was not detected in any of the samples obtained from the protective face shield of the operator prevented us from establishing the efficacy of OH˙ against pathogens of this kind. This situation can probably be explained by the protocolized screening of patients performed on a systematic basis since the World Health Organization (WHO) declared the pandemic state in 2020. Nevertheless, the efficacy of other disinfection methods against viruses with a structure similar to that of SARS-CoV-2 has been demonstrated. As an example, in a study involving the use of dry steam, Marchesi *et al*. (16) reported an efficacy of over 90% against *coronavirus OC43 *(HCoV) or human influenza virus, among others.

Finally, the implementation of phase Ib of the study, which aimed to evaluate the efficacy of OH˙ disinfection against RNA viruses such as SARS-CoV-2 and DNA viruses exclusively in the air of the dental clinic, was not considered to be necessary, since negative readings were obtained for all the samples from the protective face shield of the operator, which are the samples closest to the patient and receive the greatest impact of aerosols.

In contrast to the other abovementioned disinfection strategies, OH˙ based technology has not been tested against SARS-CoV-2 in the hospital setting. In this regard, Ge *et al*. (17) evaluated the disinfection procedures used in the Intensive Care Unit of Zhejiang University Hospital (China), where three SARS-CoV-2 patients were admitted. Disinfection of the air was carried out by means of a constant plasma flow combined with ultraviolet radiation, while surface disinfection was done with chlorinated towels applied 6 times a day. Surface sampling was performed with swabs two hours after disinfection, and sample testing was made using qRT-PCR. In only two out of 105 samples was SARS-CoV-2 RNA detected, leading the authors to conclude that the global disinfection measures adopted were effective in reducing the risk of cross-infection in the hospital setting.

Thus, the results of the present study suggest that the main advantage of the WellisAir Disinfection Wadu02® OH˙ generating device is that it can be used with the dentist and the patient present in the dental office, improving the quality of the room air. However, although a statistically significant decrease in the presence of certain microorganisms was observed, the magnitude of the effect was less than expected, and clinical studies are needed involving greater homogeneity of the procedures and randomization of the patients, in order to determine whether the results obtained effectively imply a decrease in the risk of cross-infection.

## Conclusions

The technology based on the release of hydroxyl radicals (OH˙) can be used in the course of clinical activity in the dental clinic, and reduces the presence of moulds and yeasts and *Staphylococcus* in the air, and of total aerobic bacteria on the surfaces of the clinic.
